# Method for Distinguishing Humans and Animals in Vital Signs Monitoring Using IR-UWB Radar

**DOI:** 10.3390/ijerph16224462

**Published:** 2019-11-13

**Authors:** Pengfei Wang, Yang Zhang, Yangyang Ma, Fulai Liang, Qiang An, Huijun Xue, Xiao Yu, Hao Lv, Jianqi Wang

**Affiliations:** Department of Medical Electronics, School of Biomedical Engineering, The Fourth Military Medical University, Xi’an 710032, China; wangpf2016@fmmu.edu.cn (P.W.); yangzhang@fmmu.edu.cn (Y.Z.); mayangyang@fmmu.edu.cn (Y.M.); liangfulai@fmmu.edu.cn (F.L.); qiang.an.903@outlook.com (Q.A.); xinyin20130419@163.com (H.X.);

**Keywords:** radar, human and animals, vital signs, health monitoring, variational mode decomposition (VMD), respiratory and heartbeat energy ratio

## Abstract

Radar has been widely applied in many scenarios as a critical remote sensing tool for non-contact vital sign monitoring, particularly for sleep monitoring and heart rate measurement within the home environment. For non-contact monitoring with radar, interference from house pets is an important issue that has been neglected in the past. Many animals have respiratory frequencies similar to those of humans, and they are easily mistaken for human targets in non-contact monitoring, which would trigger a false alarm because of incorrect physiological parameters from the animal. In this study, humans and common pets in families, such as dogs, cats, and rabbits, were detected using an impulse radio ultrawideband (IR-UWB) radar, and the echo signals were analyzed in the time and frequency domains. Subsequently, based on the distinct in-body structure between humans and animals, we propose a parameter, the respiratory and heartbeat energy ratio (RHER), which reflects the contribution rate of breathing and heartbeat in the detected vital signs. Combining this parameter with the energy index, we developed a novel scheme to distinguish between humans and animals. In the developed scheme, after background noise removal and direct-current component suppression, an energy indicator is used to initially identify the target. The signal is then decomposed using a variational mode decomposition algorithm, and the variational intrinsic mode functions that represent human respiration and heartbeat components are obtained and utilized to calculate the RHER parameter. Finally, the RHER index is applied to rapidly distinguish between humans and animals. Our experimental results demonstrate that the proposed approach more effectively distinguishes between humans and animals in terms of monitoring vital signs than the existing methods. Furthermore, its rapidity and need for only minimal calculation resources enable it to meet the needs of real-time monitoring.

## 1. Introduction

With growing interest in health and the life sciences, radar is garnering increasing interest, and is being applied in various scenarios as a non-contact vital signs monitoring method [1,2,3]. Within a home environment, radar technology has been used to monitor sudden infant death syndrome (SIDS), which is the third leading cause of infant mortality [4,5,6], to detect obstructive sleep apnea (OSA) and diagnose sleep disorders [7,8,9,10,11,12], and to measure heart rate, an essential physiological parameter that is closely related to a variety of diseases [13,14,15,16].

Despite the maturity of radar-based non-contact monitoring technology, and the beginning of a wide range of commercialization, there is still an important issue that has been neglected in the past, i.e., interference caused by family pets. Pets play an important role in many families; according to a report [17], the number of pet owners in China’s cities and towns reached 73.55 million in 2018, and the number of urban dogs and cats in the country reached 91.49 million. Analogously, according to the survey [18], 67% of United States households, or about 85 million families, own a pet; the total number of cats and dogs is 94.2 million and 89.7 million, respectively. Pets are often considered to be friends or a part of a family, and they generally live with people and participate in everyday activities [19,20]. This is particularly true in the case of guide dogs for the blind; they will not only accompany the owner during his/her daily routine and to work, but will also be present in medical settings where constant monitoring is desirable. Thus, in non-contact health monitoring conducted in the home environment and in medical settings, the impact of animals is a factor that cannot be ignored.

Because of the high sensitivity of radar, the vital signs of many small animals can also be detected, and some of them have a similar respiratory motion patterns to that of humans. Consequently, they are easily mistaken for human targets in non-contact monitoring, which can lead to erroneous health monitoring data and even alarms because of incorrect physiological parameters of the animal. It is therefore necessary to conduct a study to distinguish whether the subject is human or animal.

A few studies have been conducted on the distinction between humans and animals. Owing to the different lengths of legs and kinematic signatures of the target species, the gait characteristics extracted from micro-Doppler features have been used to distinguish between humans and animal directly [21,22], and to identify the category of the target by a classifier, such as a K-Nearest Neighbor (KNN) method [23], and deep convolutional neural networks (DCNNs) [24]. However, in most application scenarios of non-contact health monitoring, the targets are stationary, and the strategy based on Micro-Doppler motion characteristics is not feasible. Wang et al. [25] proposed a wavelet entropy-based method to distinguish between humans and dogs through a brick wall and in free space using radar with a low center frequency of 400 MHz [25]. Wavelet entropy can measure the degree of signal ordering; it has a strong correlation with the signal-to-noise ratio (SNR) of the signal. Under the radar system with a center frequency of 400 MHz, the wavelet entropy of humans and dogs differ significantly because of the strong system noise. However, for vital sign monitoring radars with high resolution and high SNR, the difference in wavelet entropy between humans and dogs is not evident. Moreover, a correlation coefficient-based method has also been proposed to distinguish between humans and dogs [26]. The correlation coefficient in [26] shows the strength and direction of the linear relationship between the target variables and others. However, similar to [25], the correlation coefficient is directly related to the noise level of the radar system. Under high SNR conditions, the difference in the parameters between humans and animals is small, and therefore, the distinguishing effect is not appropriate. 

For some small animals, such as rabbits, we can distinguish them from humans directly by their respiratory frequency range [27]. However, as more common pets, i.e., cats and dogs, have a similar respiratory rate range to humans [28,29], it is difficult to separate them from humans by relying on methods that are based on respiratory rate range. Another important physiological parameter is the heart rate, which can also be measured using a radar. Animal heart rate ranges have a large intersection with the human heart rate range [30,31]. Further, when some diseases occur, the human heart rate will rise and be close to the animal’s heart rate. Therefore, it is unreasonable to distinguish between humans and animals by the criterion of heart rate.

Because of the differences in the body structure of humans and animals, particularly for cardiopulmonary organs, we propose to distinguish humans from animals by measuring the contribution rate of the respiratory and heartbeat signals in the vital signs detected by radar. In order to acquire respiratory and heartbeat signals, the echo data after preprocessing should be processed by mode decomposition algorithms. Although discrete Fourier transform can convert time-domain signals into frequency domain signals, we still need a better algorithm to obtain weak information [32,33]. Because the variational mode decomposition (VMD) algorithm is efficient and stable in non-stationary signal processing [34], it is widely used in mechanical fault diagnoses [35,36], biomedical signal processing [37,38], and remote sensing signal analysis [39,40]. Therefore, it is used to extract respiratory and heartbeat signals.

In this study, we investigated the vital signs of dogs, cats, rabbits, and humans through an impulse radio ultrawideband (IR-UWB) radar, and we propose a scheme for distinguishing between humans and animals. In our scheme, after the target echo signal is received by the radar, the background noise is removed by subtracting the mean value, and the direct-current (DC) components are suppressed by an FFT-based (fast Fourier transform) FIR (finite impulse response) filter. The target is then located and initially identified by energy threshold conditions. Then, a VMD algorithm is used to decompose the signal into multiple variational intrinsic mode functions (VIMFs), from which the respiration and heartbeat signals are selected. Finally, humans and animals are identified using our proposed respiratory and heartbeat energy ratio (RHER) index, which is calculated using the VIMFs selected based on human respiration and heart rate range.

The remainder of this article is organized as follows. Section 2 presents the signal processing method. Section 3 presents the architecture of the IR-UWB radar and outlines the experiments conducted on humans and animals. Section 4 describes the application of the proposed approach to realize the distinction between humans and animals. Finally, Section 5 presents concluding remarks.

## 2. Methods

In this section, we introduce the signal processing methods and proposed discriminant indicators used in this paper.

### 2.1. Data Acquisition and Signal Preprocessing

**Figure 1:**
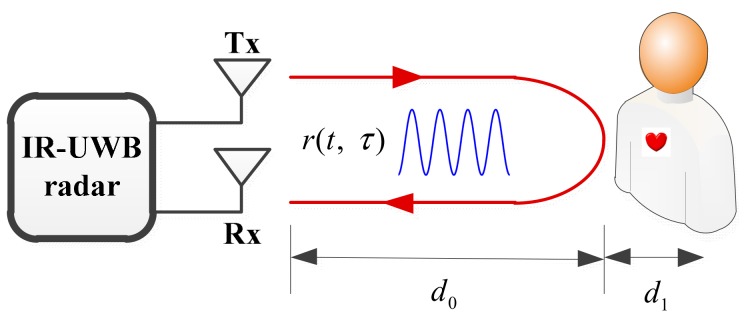
Schematic diagram of the radar detection principle. The time-of-flight of the pulse $\tau_{0}$ depends on antenna distance, $d_{0}$.

The schematic of the radar detection principle is illustrated in Figure 1. Tx is a transmitting antenna, and Rx is a receiving antenna. For IR-UWB radar systems, when the transmitted pulse hits the human target, part of it is reflected, owing to the high reflectivity of the body to radio frequency (RF).

The time-of-flight (ToF) of this pulse is denoted by $\tau_{0}$; it depends on the antenna distance, $d_{0}$. $d_{1}$ is the displacement caused by breathing and heartbeat. Owing to respiration and heartbeat motions, the chest expands and contracts periodically, and therefore, the distance d(t) changes regularly around the nominal distance $d_{0}$. For vital signs monitoring, d(t) can be expressed as

(1) $$d(t)=d_{0}+d_{1}=d_{0}+m_{b}\sin(2\pi f_{b}t)+m_{h}\sin(2\pi f_{h}t),$$

where $m_{b}$ and $m_{h}$ are the amplitudes caused by the respiration and the heartbeat, respectively, $f_{b}$ and $f_{h}$ are the frequencies of respiration and heartbeat, respectively.

When multiple channels exist, the received signal can be represented as

(2) $$r(t,\;\tau )\;=\sum_{i}A_{i}p(\tau -\tau_{i})\;+\;A_{T}p(\tau -\tau_{d}(t)),$$

where $p\left(t\right)$ is a normalized received pulse, $A_{i}$ is the amplitude of each multipath component, while $\tau_{i}$ is the corresponding delays. $A_{T}$ is the amplitude of the pulse reflected owing to the movement of the chest.

The sampled radar echo signal r(t, τ) is stored as a matrix S(x,n) of size X×N. The axis X represents the detection distance, called "fast time", and the unit is ns. The relationship between the round-trip range “X” and fast-time “t” is t=2R/c, where c is the speed of flight. It is generally converted to distance according to its correspondence with the round-trip range, and the unit is m. The axis N represents the detection time, which is called “slow time”, and the unit is s(seconds) [41].

The clutter caused by stationary objects, such as walls and furniture, generally leads to a baseline drift in those signals along the slow time dimension. To remove the clutter of stationary objects, the average of all waveform values with slow time is subtracted by a sliding window, and a finite impulse response (FIR) filter is adopted along the slow time dimension for suppressing noise [42]. 

DC removal removes the DC component and baseline drift. The DC removal is given by

(3) $$S_{DC}(x,n)=S(x,n)-\frac{1}{Q}\sum_{n=x}^{x+Q-1}S(x,n),$$

where $S_{DC}(x,n)$ is the radar data after background removal, and Q is the signal length of the sliding window.

The cut-off frequency of the low-pass filter is chosen to be 5 Hz to filter out high-frequency noise and retain respiratory and heartbeat signals. It can be determined by:

(4) $$S_{LP}(x,n)=S_{DC}(x,n)*H(t)$$

where H(t) is the impulse response function of the finite impulse response (FIR) filter [42].

### 2.2. Energy Indicator

To facilitate the distinction between people and small animals, the energy index of targets is used for initial discrimination. Owing to the high resolution of the non-contact monitoring radar [43], the received echo signal generally has a high SNR after signal preprocessing. Therefore, it is the most convenient and effective method to locate the target and preliminarily judge the category of the target (small animals or humans) based on the energy of the signal [41]. For a row of the target data $S_{LP}(x,n)$, a 1×N signal $s_{x}(n)$, the energy indicator can be easily calculated as

(5) $$E_{x}=s_{x}{\left(1\right)}^{2}+s_{x}{\left(2\right)}^{2}+\ldots +s_{x}{\left(N\right)}^{2}=\sum_{n=1}^{N}s_{x}{\left(n\right)}^{2},$$

where x indicates the position interval of the target, $\left\{x\right\}=\left\{1,2,\ldots ,X\right\}$, and X corresponds to the set radar detection range. 

The energy indicator of $S_{LP}(x,n)$ is $\left\{E\right\}=\left\{E_{1},\ldots E_{x},\ldots ,E_{X}\right\}$. By searching for the maximum value $E_{x}$ of E, we can determine the location of the target by index x, where $E_{x}$ is the energy of the target, and the vital signs signal of target is $s_{x}(n)$.

**Figure 2:**
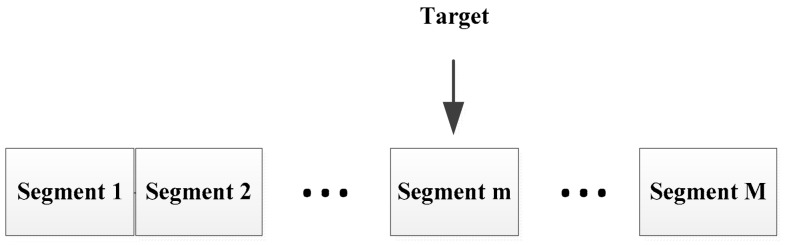
Detection range segmentation process; the detection range is divided into M segments.

Taking the influence of distance into account, we divide the detection range into M segments, as shown in Figure 2.

In every segment, multiple human targets are detected and their average energy is calculated as the human target energy standard, which is a collection of constants $\left\{E_{m,human}\right\}=\left\{E_{1,human},\ldots E_{m,human},\ldots ,E_{M,human}\right\}$, where $\left\{m\right\}=\left\{1,2,\ldots ,M\right\}$ is the serial number of the segments, which is determined by the position interval i.

(6) $$m=\left[i\frac{M}{I}\right]+1,$$

where the symbol [] represents the rounding down function. Parameter $\alpha_{E}$ is the energy ratio of the target to the energy standard of the human.

(7) $$\alpha_{E}=\frac{E_{m,human}}{E_{i}}.$$

Here, the threshold is set to 10. When $\alpha_{E}$ is below the threshold, the target is judged to be human and the signal is processed in the next step. When the $\alpha_{E}$ is above the threshold, the target is judged to be an animal.

### 2.3. Variational Mode Decomposition

VMD is a new adaptive decomposition method based on Wiener filtering and Hilbert transform [34]. By searching for the optimal solution of the constrained variational model, the original signal can be decomposed into a set of VIMF components with sparse characteristics. Each mode is compact around a center pulsation $\omega_{k}$, and its bandwidth is estimated using $H^{1}$ Gaussian smoothness of the shifted signal. The VMD is written as a constrained variational problem [34]:

(8) $$\underset{\left\{u_{k}\right\},\left\{\omega_{k}\right\}}{\min}\left\{\sum_{k=1}^{K}{\left\|\partial t\left[\left(\delta (t)+\frac{j}{\pi t})*u_{k}(t\right)\right]e^{-j\omega kt}\right\|}_{2}^{2}\right\}\;s.t.\;\sum_{k=1}^{K}u_{k}=f,$$

where K is the number of VIMFs, f is the input signal, and $f=s_{x}(n)$; furthermore, $\left\{u_{k}\right\}=\left\{u_{1},u_{2},\cdots ,u_{K}\right\}$ and $\left\{\omega_{k}\right\}=\left\{\omega_{1},\omega_{2},\cdots ,\omega_{K}\right\}$ are shorthand notations for the set of all modes and their center frequencies, respectively.

Equation (8) can be addressed by introducing a quadratic penalty and Lagrangian multipliers. The augmented Lagrangian is given as follows [34,35]:

(9) $$L(\left\{u_{k}\right\},\left\{\omega_{k}\right\},\lambda )=\alpha \sum_{k=1}^{K}{\left\|\partial_{t}\left[\left(\delta (t)+\frac{j}{\pi t})*u_{k}(t\right)\right]e^{-j\omega_{k}t}\right\|}_{2}^{2}+{\left\|f(t)-\sum_{k=1}^{K}u_{k}(t)\right\|}_{2}^{2}+\langle \lambda (t),f(t)-\sum_{k=1}^{K}u_{k}(t)\rangle$$

in which α denotes the balancing parameter of the data-fidelity constraint. Equation (9) is then solved with the alternate direction method of multipliers (ADMM) [44]. All the modes gained from solutions in the spectral domain are written as follows:

(10) $${\hat{u}}_{k}^{}(\omega )=\frac{\hat{f}(\omega )-\sum_{i\neq k}{\hat{u}}_{i}^{}(\omega )+\frac{\hat{\lambda }(\omega )}{2}}{1+2\alpha {\left(\omega -\omega_{k}^{}\right)}^{2}},$$

where $\omega_{k}$ is computed at the center of gravity of the corresponding mode’s power spectrum. Thus, Wiener filtering is embedded into the VMD algorithm, which makes it much more robust to sampling and noise.

(11) $$\omega_{k}^{}=\frac{\int_{0}^{\infty }\omega {\left|{\hat{u}}_{k}^{}\right|}^{2}d\omega }{\int_{0}^{\infty }{\left|{\hat{u}}_{k}^{}\right|}^{2}d\omega }$$

The details of the complete VMD algorithm can be found in [34].

Applying the VMD algorithm to the received data after pre-processing, the signal can be decomposed into multiple VIMFs,$\left\{u_{k}\right\}=\left\{u_{1},u_{2},\cdots ,u_{K}\right\}$. According to the frequency range of the breathing and heartbeat, $u_{r}$ and $u_{h}$, representing respiration and heartbeat, can be selected. According to previous experiments, the respiratory and heartbeat components of the human signal are VMF1 and VMF2, respectively. 

### 2.4. Respiratory and Heartbeat Energy Ratio

To effectively distinguish between humans and animals, we propose a new indicator, RHER, which reflects the different contribution rates of respiration and heartbeat in vital signs. By comparing the anatomical maps of human and animals, we believe that the difference in body structure is one of the reasons for the differentiation of this parameter [45,46,47,48]. Compared with humans, the hearts of dogs and cats are located close to the front ribs of the body; the fat and muscle layer between the skin and the heart are thin, which may cause heartbeat movement to be detected more evidently than with humans. 

The lungs and heart are critical organs of respiratory and heartbeat movements, and organ mass is one of the essential parameters commonly used in medical research. According to [49,50], for normal adult men and women, the average weight of the lungs and heart are found and shown in Table 1, and the lung-to-heart weight ratio (LHWR) was calculated to be 2.99 and 3.15. At the same time, according to [51,52,53], the weight of the organs of dogs, cats, and rabbits can also be found; the calculated LHWRs are listed in Table 1. In the table, it can be seen that the LHWRs of male and female dogs are 1.12 and 1.13, and those of cats and the rabbits are 1.5 and 1.49, respectively. The LHWRs of animals (dogs, cats, and rabbits) evidently differ from those of humans. 

It is widely known that the weight of the organ is directly and positively related to the volume. For humans and animals, complex conductivity or complex permittivity can be well represented by the Cole-Cole equation [54,55,56,57]. Irrespective of the small differences in the dielectric parameters of humans and animals, the volume of the heart and lungs directly determines the radar-cross section (RCS) of their organs. Therefore, the distinctive body structure between humans and animals can directly lead to differences in the strength of the respiratory and heartbeat components in vital signs. By measuring the contribution rate of respiratory and heartbeat signals in vital signs, humans and animals can be distinguished.

Because the target of non-contact monitoring is a human, this study aimed to rule out the interference of animals during human health monitoring. Therefore, we considered the law of human vital signs as the criterion by which to explore parameters that are significantly different from animals.

After the target passes the initial determination, the VMD is applied, and the VIMFs $u_{r}$ and $u_{h}$ based on the frequency range of the respiration and heartbeat for humans can be selected. Then, the RHER index can be calculated by

(12) $$RHER=\frac{\sum_{t}{\left(u_{r}(t)\right)}^{2}}{\sum_{t}{\left(u_{h}(t)\right)}^{2}}.$$

In the experiment, we found that the ratio of the respiratory energy to the heartbeat energy of animals is significantly smaller than that of humans, which means that the RHER of humans has a significant difference compared with that of animals. 

**Figure 3:**
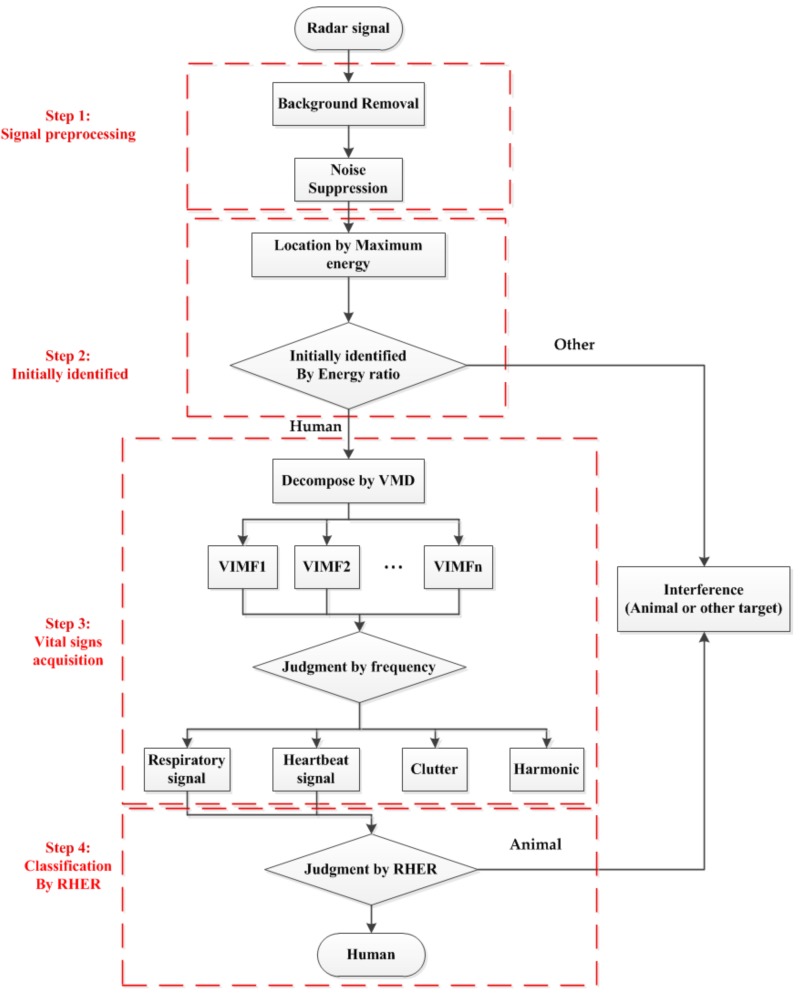
System diagram of signal processing, which can be divided into 4 steps.

The classification process of the target can be divided into four steps: signal preprocessing, initially identification, vital signs acquisition, and target classification using RHER. A block diagram is shown in Figure 3.

## 3. Architectures and Experiments

In this section, we present the architecture of the IR-UWB radar and experiments on humans, dogs, cats, and rabbits.

### 3.1. IR-UWB Radar System

For vital sign monitoring, high resolution is necessary. In previous studies, radar systems used for vital sign monitoring generally have high center frequencies, except for through-wall detection [1,2,43]. 

**Figure 4:**
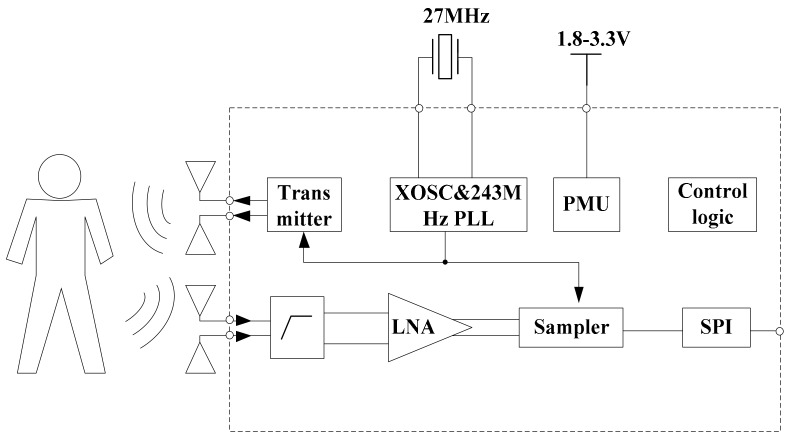
Block diagram of the radar sensor SoC.

In this study, a typical IR-UWB radar system on chip (SoC) called X4M02 (XeThru, Novelda, Norway) was utilized. Figure 4 shows that there are many components in the system architecture. The TX transmits pulses with an interval determined by the pulse repetition frequency (PRF); the RX receives the echo signal after the delay corresponding to the round-trip ToF to the target and return, by which a frame, i.e., a digitized representation of the time-domain range profile, is created.

The bandwidths of the transmitter are 1.4 and 1.5 GHz, and the center frequencies of the sensor are 7.29 and 8.748 GHz. The mode we used in this study is the 7.29 center frequency with a 1.4 GHz bandwidth. The receiver samples of the reflected signal are at 23.328 GS/s, and it can cover a 9.9 m consecutive range. Here, the detection distance range we set is 5 m. The radar communicates with a computer via a USB cable and receives raw radar data in real-time. More information about the radar sensor can be found in [43].

### 3.2. Experiments

This study involved 10 healthy humans aged between 22 and 35 years. All volunteers were informed about the experimental content and signed an informed consent form. 

This study also involved five healthy dogs aged about 1 year and weighing between 10 kg and 22 kg, five healthy cats aged between 1 and 3 years and weighing between 1 kg and 3.5 kg, and five healthy rabbits aged between 3 and 5 months and weighing between 2 kg and 3 kg. All animals were provided by the Fourth Military Medical University, and the Experimental Animal Welfare and Ethics Committee of the Fourth Military Medical University approved all experimental procedures (No. 20190901).

#### 3.2.1. Human Vital Signs Detection

**Figure 5:**
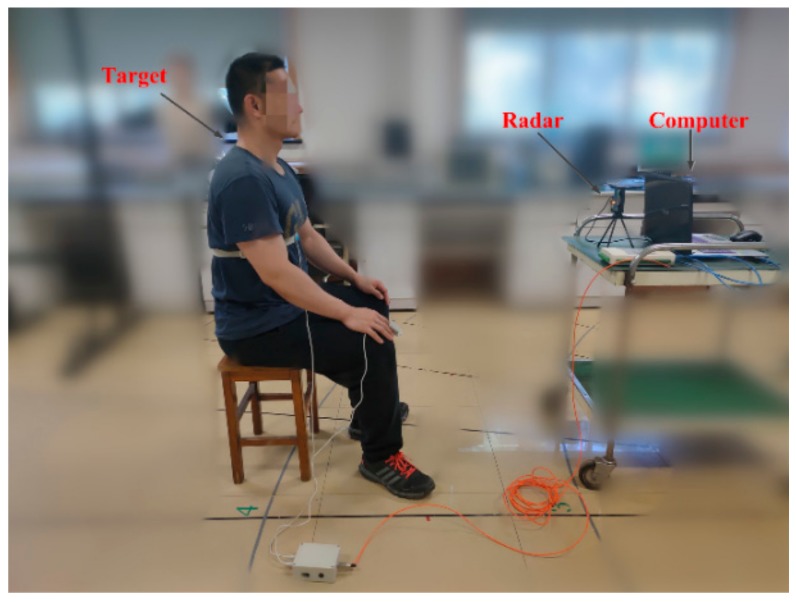
Experimental environment for human vital signs detection.

In this part, the 10 human volunteers served as the targets for radar detection; the experimental scene is shown in Figure 5. The radar connects to the computer through a USB interface to collect real-time data; the target is about one meter away from the radar sensor. 

**Figure 6:**
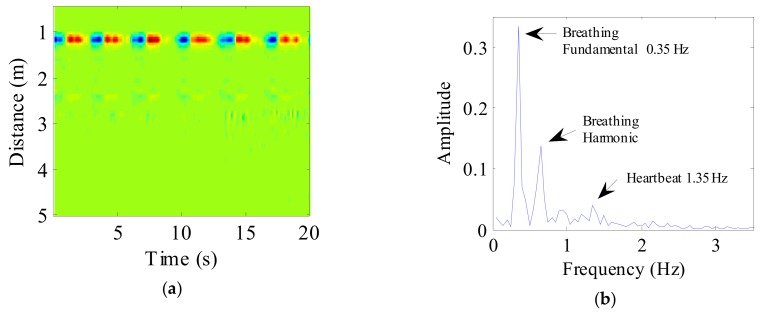
(a) The radar echo data after signal preprocessing, where the abscissa represents time and the ordinate represents distance; (b) The spectrum of human vital sign signal.

A 20 s radar echo signal received from a human volunteer following background removal and DC component suppression is shown in Figure 6a. The target at a distance of 1 m can be seen clearly. Simultaneously, evident periodic breathing signals can also be seen. The spectrum obtained following FFT application to the target signal is shown in Figure 6b, where the frequency of human breathing is very evident in the spectrum, although the heartbeat component is very weak.

**Figure 7:**
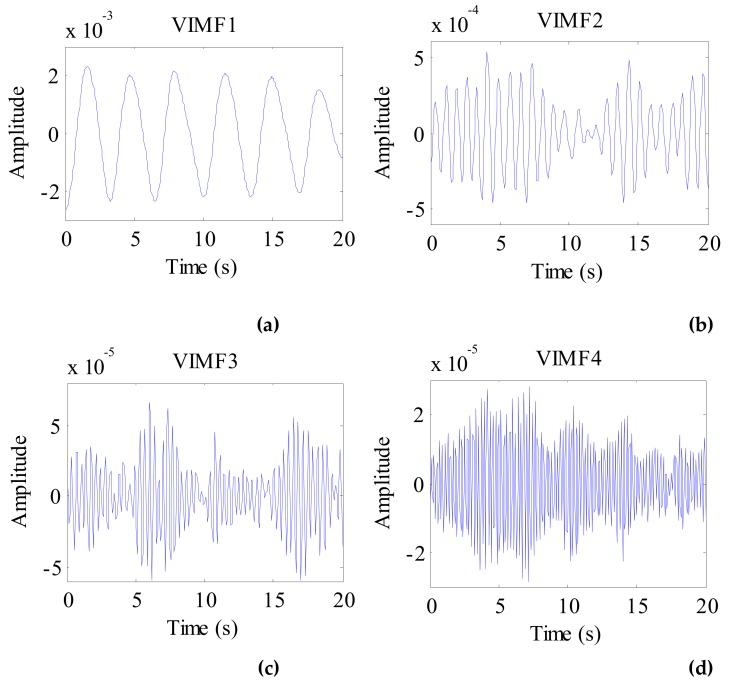
The VIMFs of human vital sign signal decomposed by VMD. (**a**) The waveform of VIMF1; (**b**) The waveform of VIMF2; (**c**) The waveform of VIMF3; (**d**) The waveform of VIMF4.

**Figure 8:**
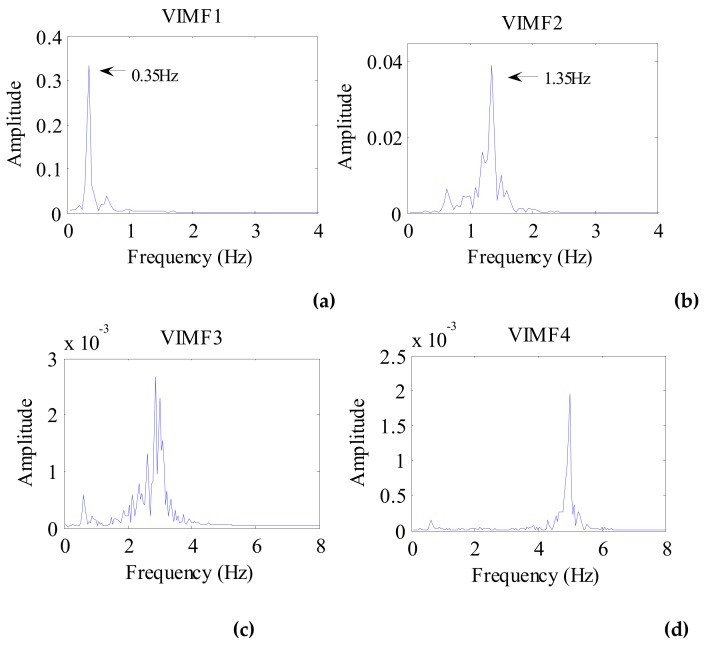
The spectrum of human VIMFs obtained by FFT. (**a**) The spectrum of VIMF1; (**b**) The spectrum of VIMF2; (**c**) The spectrum of VIMF3; (**d**) The spectrum of VIMF4.

The data after signal preprocessing is considered as input parameters into the VMD algorithm. The number of modes is set to 4. The balancing parameter is set to 9000, and the tolerance is set to 10^−6^. The VIMFs of human vital signs acquired by VMD are shown in Figure 7. A clear respiratory motion waveform can be seen in VIMF1, while the heartbeat component is represented by VIMF2. The components in VIMF3 and VIMF4 are the harmonics of the breathing and heartbeat components and clutter. The spectra of the VIMFs following the application of FFT are shown in Figure 8. From the figure, we can see that VMF1 is the respiratory component of the human, while VMF2 comprises the heartbeat component. In this example, the respiration frequency is 0.35 Hz, which is the same as that obtained by direct processing of the waveform; the heartbeat frequency is 1.35 Hz. In Figure 7 and Figure 8, the ordinate axis represents the amplitude of the signal; we can see that the amplitude of the respiratory component is much larger than that of the heartbeat component.

#### 3.2.2. Dog Vital Signs Detection

**Figure 9:**
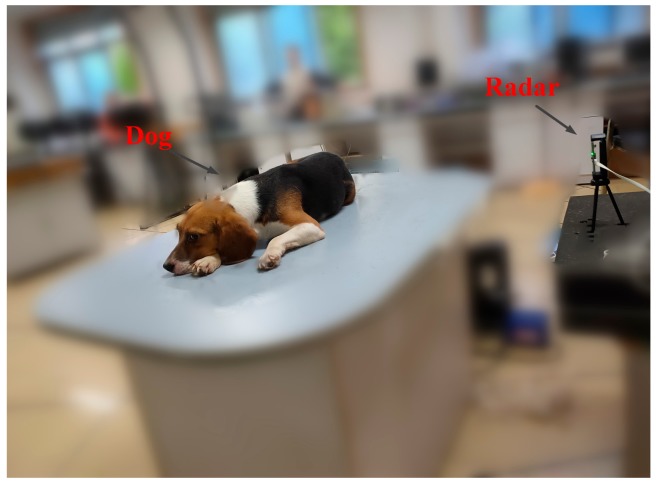
Setup for dog vital signs detection.

Five dogs were used as targets in this experiment. The experimental environment is shown in Figure 9. The dog remained calm at a distance of 1 m from the radar.

**Figure 10:**
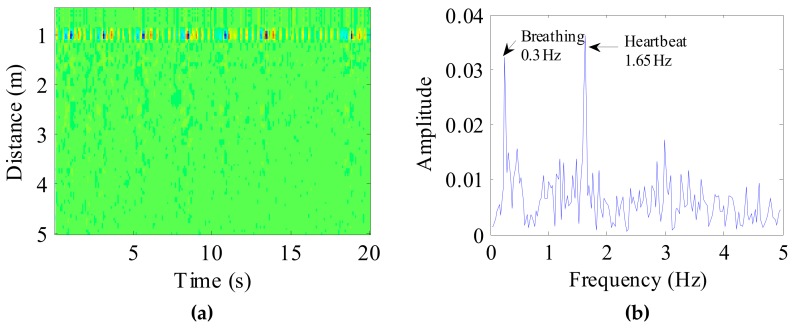
(**a**) Received radar echo data after signal preprocessing, where the abscissa represents time and the ordinate represents distance; (**b**) The spectrum of the dog vital sign signal.

After pre-processing, a 20s radar echo data was as shown in Figure 10a. In the figure, the location of the dog and the respiratory cycle of the target can be seen directly. According to the position of the target, the dog vital sign signal was selected. Applying FFT to the target signal, the spectrum of the dog vital sign signal is shown in Figure 10b. In the figure, we can clearly see the target’s breathing and heart rate.

**Figure 11:**
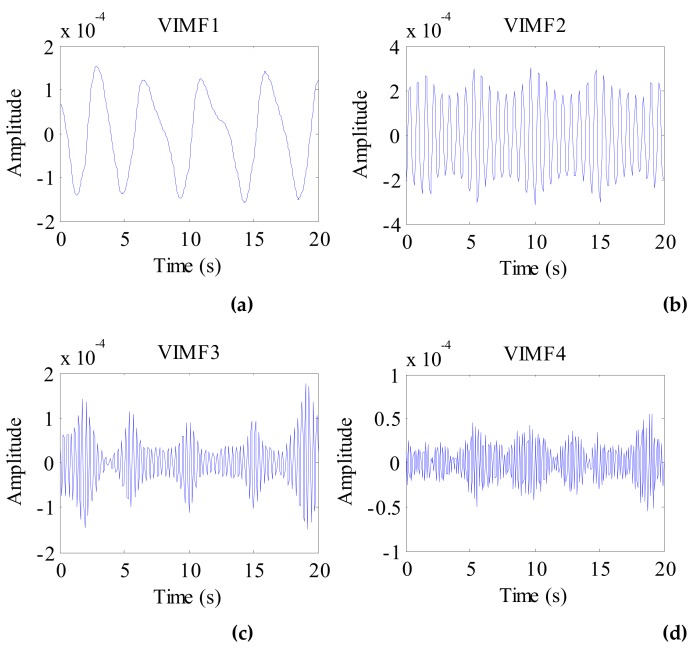
The VIMFs of dog vital sign signal decomposed by VMD. (**a**) The waveform of VIMF1; (**b**) The waveform of VIMF2; (**c**) The waveform of VIMF3; (**d**) The waveform of VIMF4.

**Figure 12:**
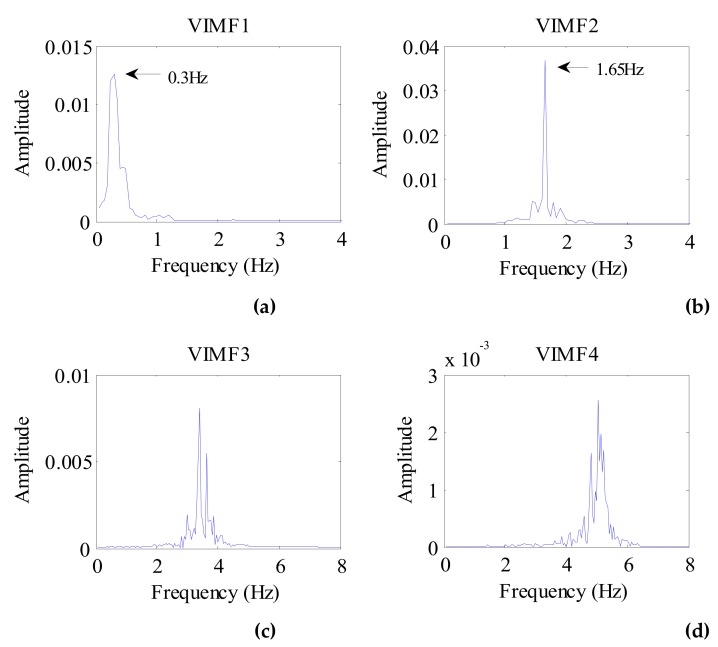
The spectrum of dog VIMFs obtained by FFT. (**a**) The spectrum of VIMF1; (**b**) The spectrum of VIMF2; (**c**) The spectrum of VIMF3; (**d**) The spectrum of VIMF4.

Considering the preprocessed signal as the input of the VMD algorithm and keeping the other parameters the same, the VIMFs of the dog were obtained and are shown in Figure 11. The dog’s breathing and heartbeat components are well decomposed in VMF1 and VIMF2, respectively. The harmonics of the breathing and heartbeat components and clutter are decomposed in VIMF3 and VIMF4. Applying FFT to VIMFs, the resulting spectrum is shown in Figure 12. As shown in the figure, the dog’s breathing frequency is 0.3 Hz and the heartbeat frequency is 1.65 Hz, both of which are in the respiratory and heartbeat frequency range of the human body; however, the heart rate is higher than that of the average of the human. At the same time, we can see that the amplitude of the heartbeat component is stronger than the respiratory component, which is different from the human target signal.

#### 3.2.3. Cat and Rabbit Vital Signs Detection

Five cats and five rabbits were used as targets in this experiment. The experimental environment was the same as that for dogs. The cats and rabbits remained calm or slept at a distance of 1 m from the radar.

**Figure 13:**
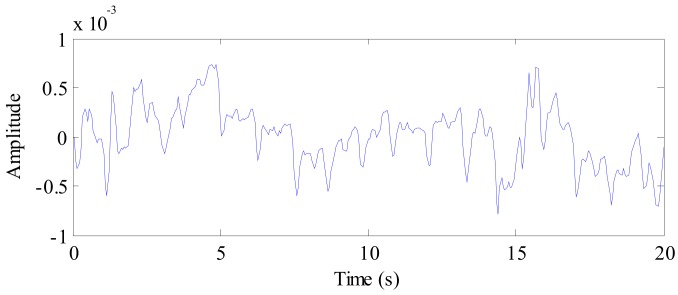
The waveform of the cat vital sign signal after background removal.

A 20 s vital signal of a cat following background removal and target location is shown in Figure 13. In the figure, we can see the periodic breathing waveforms and the smaller waveforms of the heartbeat; simultaneously, the DC component of the signal can be seen clearly. Therefore, the DC component is also suppressed during signal preprocessing.

**Figure 14:**
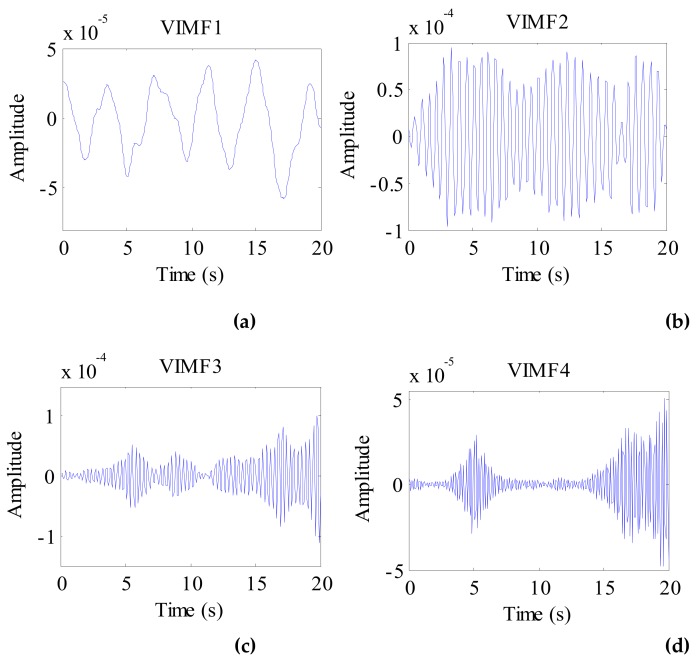
The VIMFs of cat vital sign signal decomposed by VMD. (**a**) The waveform of VIMF1; (**b**) The waveform of VIMF2; (**c**) The waveform of VIMF3; (**d**) The waveform of VIMF4.

**Figure 15:**
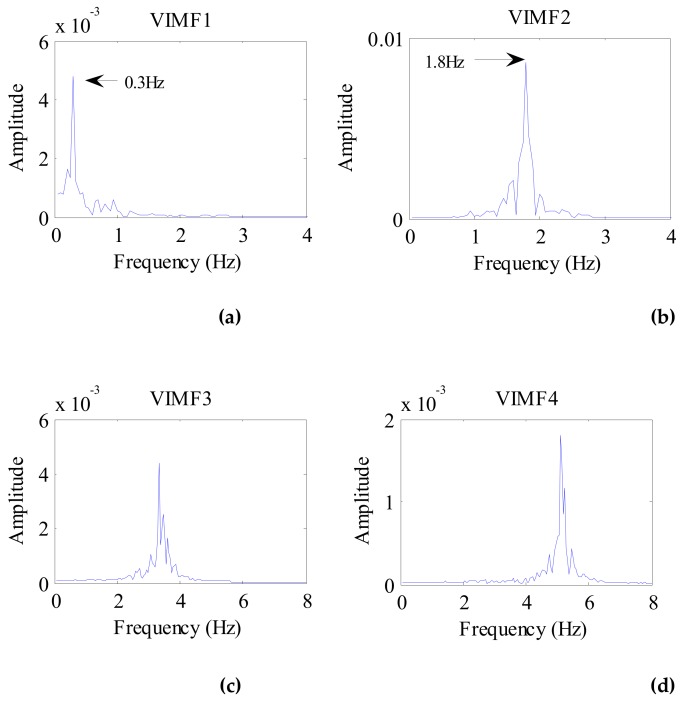
The spectrum of cat VIMFs obtained by FFT. (**a**) The spectrum of VIMF1; (**b**) The spectrum of VIMF2; (**c**) The spectrum of VIMF3; (**d**) The spectrum of VIMF4.

Applying the VMD algorithm to the preprocessed signal of the cat, the VIMFs of the cat are shown in Figure 14. In the figure, the respiratory component is represented by VMF1 and the heartbeat component is represented by VIMF2. VMF3 and VMF4 are harmonics and clutter of the respiratory and heartbeat components. The spectrums of VIMFs obtained by an FFT are shown in Figure 15. In the figure, we can see that the frequency of VIMF1 is 0.3 Hz, and the frequency of VIMF2 is 1.85 Hz, which are similar to a healthy human’s breathing and heart rate. Similar to the dog’s vital sign signals, the amplitude of the VIMF2 of the cat is also stronger than that of the VIMF1 component.

**Figure 16:**
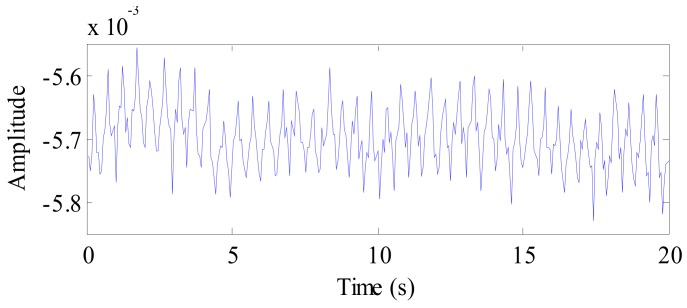
The waveform of the rabbit vital sign signal after background removal.

For the rabbit as a target, the signal received by radar detection is shown in Figure 16. Owing to the high sensitivity of the radar, the rabbit vital signs are well detected. Compared to the weak vital signs of the rabbit, the visible DC component that can be found in the figure is evident, which is caused by the movement of the rabbit.

**Figure 17:**
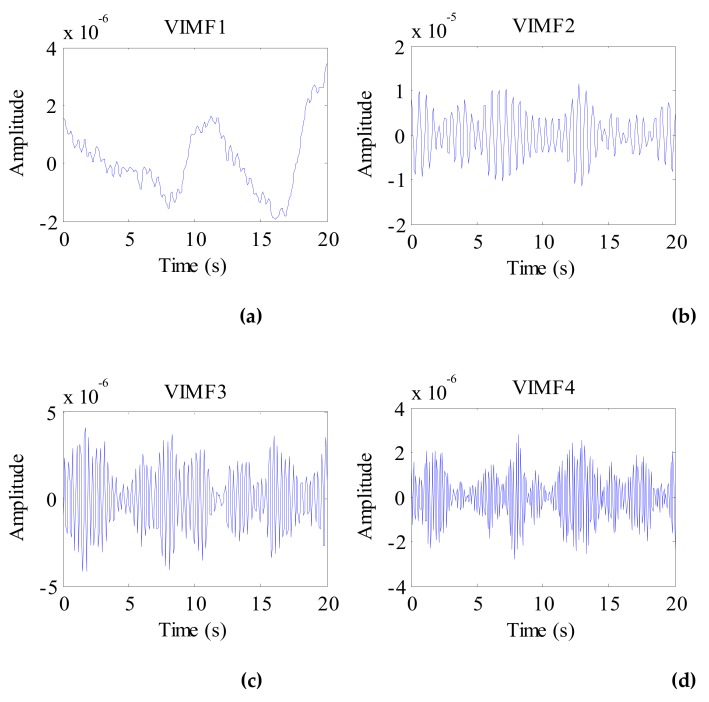
The VIMFs of the rabbit vital sign signal decomposed by VMD. (**a**) The waveform of VIMF1; (**b**) The waveform of VIMF2; (**c**) The waveform of VIMF3; (**d**) The waveform of VIMF4.

**Figure 18:**
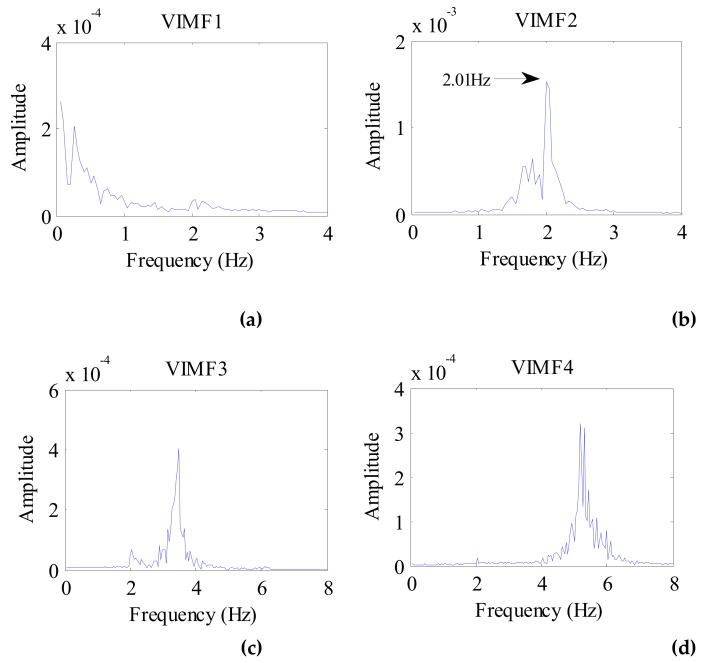
The spectrum of rabbit VIMFs obtained by FFT. (**a**) The spectrum of VIMF1; (**b**) The spectrum of VIMF2; (**c**) The spectrum of VIMF3; (**d**) The spectrum of VIMF4.

The VIMFs of the rabbit vital signs decomposed by the VMD algorithm are shown in Figure 17, and the spectrums of the rabbit VIMFs acquire by FFT are shown in Figure 18. In contrast to other targets (human, dog, and cat), the rabbit has a higher respiratory rate. Therefore, following decomposition by VMD, the VMF1 mainly includes low-frequency body motion components, while VIMF2 consists of respiratory components. VIMF3 represents the heartbeat component of a rabbit, and the components in VIMF4 represent the harmonics of rabbit breathing and heartbeat. Because the background was removed, and the low frequency DC component was suppressed during the preprocessing stage, the energy of VMF1 is much smaller than VMF2. In this condition, the RHER index has the same characteristics as those of the dog and cat, i.e., much lower than those of a human.

## 4. Target Category Judgment

In this section, the applications of the energy index and the RHER index are described to realize the distinction between humans and animals. 

### 4.1. Distinguishing Based on Energy

Because the body motion amplitude of rabbits and cats caused by respiration and heartbeat is smaller than that of a human, it would appear that the most intuitive way to distinguish between different targets is to judge the energy of the signals received. However, because of the individual differences in objectives, this approach has considerable limitations, such as not being applicable to dogs. Therefore, in this study, this method was only used for the initial determination because of its simplicity and efficiency.

**Figure 19:**
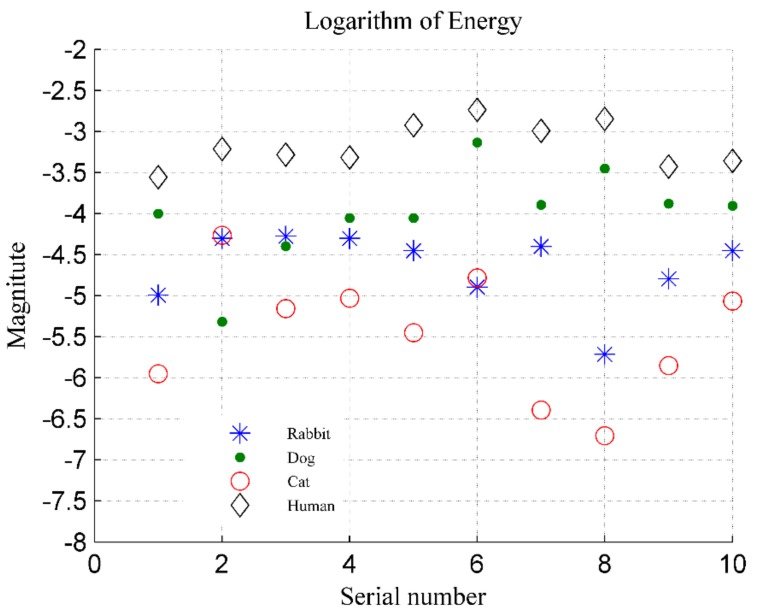
Energy indicators for four categories of targets, where the ordinate represents the logarithm to base 10 of energy.

The logarithms to the base 10 of the energy indicator of different targets (human, dog, cat, and rabbit) are shown in Figure 19. For the human targets, the data from 10 volunteers is represented by the abscissa axis, and their energy index is represented by the ordinate axis. For animals (dog, cat, and rabbit), the data measured in two dates is represented by the abscissa axis, and their energy indicator is represented by the ordinate axis. In the figure, we find that the energy of humans is much higher than that of rabbits and cats, and most data of rabbits and cats meets the threshold conditions. However, there are still some data from cat and rabbit which cannot be accurately discriminated based on the energy indicator. In the figure, the magnitudes of the cats and rabbits are sometimes higher than −4.5, and the magnitudes of humans are close to −4.5; the energy ratio $\alpha_{E}$ of the cat and rabbit will be lower than the threshold, and they would be misjudged as a human target.

Further, the distinction between dogs and humans is not useful, because the dog’s energy is sometimes higher than that of the human, such as the target data with the serial number 6 and 8 in Figure 19. In addition, individual differences in detection targets also need to be considered. The cats used in this experiment are in a relatively normal weight range, and their energy is small; but in practice, some cats are often overweight, such as orange cats and British shorthair cats, and their energy indicator would be higher. Therefore, a more effective method of discriminating is needed.

### 4.2. Distinguishing Based on RHER Index

According to the process in Figure 3, after the initial determination based on the energy indicator, human VIMFs are obtained by the VMD algorithm. In this paper, VIMF1 represents the respiratory component of the human, VIMF2 represents the heartbeat component; these values are selected as $u_{r}$ and $u_{h}$, respectively.

**Figure 20:**
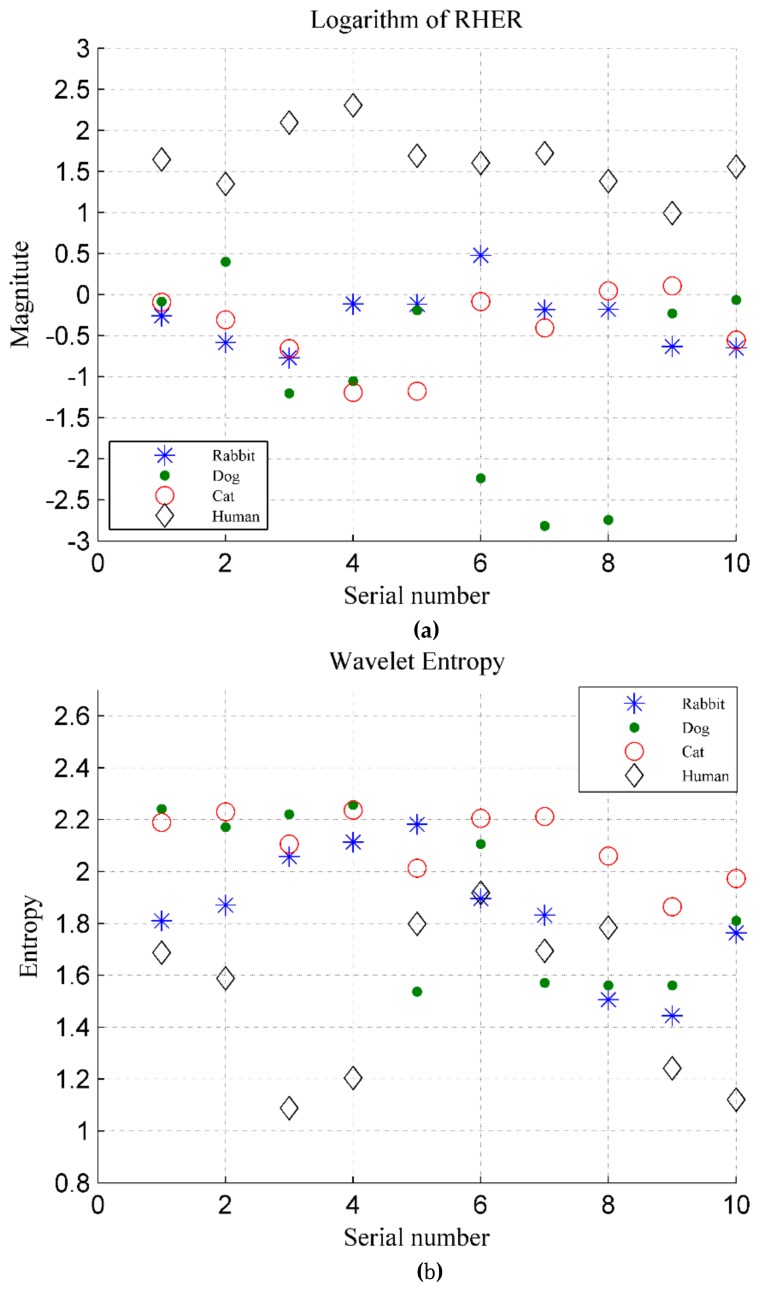
(**a**) RHER index for four categories of targets, where the ordinate represents logarithm to base 10 of RHER; (**b**) Wavelet entropy for four categories of targets.

The RHER index is calculated by using Equation (12), and the logarithm to the base 10 of the RHER is shown in Figure 20a. For comparison, the discrimination based on wavelet entropy in [25] is shown in Figure 20b, in which a Daubechies wavelet, i.e., the db7 basis function, is applied. Similar to Figure 19, for the human target, the abscissa axis represents the 10 volunteers, and the ordinate axis represents their energy index. For animals, the number on the abscissa axis represents the samples of five animals measured in two dates, and the ordinate axis represents the results of samples.

Figure 20a shows that the RHER index of different targets has evident differences. Because the VMF1 indicates the movement of body rather than breathing, the RHER index of individual rabbits is high than those of other animals owing to incomplete suppression of low frequency interference during the preprocessing. The RHER of dog and cat is much smaller because the heartbeat detected by radar has a high contribution rate in vital signs.

Wavelet entropy was used to measure the order of the signal. In this experimental system, owing to the higher SNR, the signals of animals and people were all apparent and showed reasonable periodicity. Figure 20b shows that the method based on wavelet entropy does not perform ideally. Although the wavelet entropy of humans is slightly lower than that of the animals, it can not distinguish between humans and animals well, particularly for dogs and rabbits.

## 5. Conclusions

In this study, we investigated animal interference of human vital signs monitoring within a home environment. Common house pets, such as dogs, cats, and rabbits, were considered as targets and detected in free space. During the analysis of the animals’ vital signs, it was verified that the respiratory rate and heartbeat rates of dogs and cats are similar to that of human beings; however, this is not the case for the rabbit.

This paper described the use of quantitative parameters in the analysis of the returned signals obtained by an IR-UWB radar to investigate the differences between human targets and animals. Owing to the findings in the experiment that the body motion amplitude of the animal is smaller than that of human, energy indicators were used to make initial judgments on people and animals, by which interference caused by a small animal can be easily eliminated. 

For further distinguishing between humans and animals, the RHER index was proposed in this paper. After the signal preprocessing, the $u_{r}$ and $u_{h}$ selected from VIMFs based on the frequency range of the human body’s respiration and heartbeat were obtained by the VMD algorithm and used to calculate the RHER index. RHER can reflect the contribution rate of breathing and heartbeat in the detected vital signs; the difference in RHER between humans and animals is caused by the distinct structures of the body. The findings of this study facilitate the differentiation of human targets and animal targets through the use of radar.

The experimental results show that the proposed scheme can more effectively distinguish between humans and animals in human vital signs monitoring than existing methods. Moreover, the proposed approach consumes less computing resources and can meet the needs of real-time monitoring. The improved performance of the radar system allows the radar sensor to better meet the requirements of practical applications and will help to accelerate its commercialization.

In future work, the signals of humans and animals when people and pets sleep in a bed together will need to be separated, and simultaneous monitoring of humans and animals realized.

## Figures and Tables

**Table 1:** The organ weights of humans and animals.

Target		Weight (kg)	Organ Weight (g)		LHWR
			Lungs	Heart	
Human	M	75	1136	380	2.99
	F	50	797	243	3.28
Dog	M	9.89	86.13	76.86	1.12
	F	8.74	77.63	68.52	1.13
Cat	M&F	1.542	15	10	1.50
Rabbit	M&F	2.587	13.72	9.23	1.49

M: male; F: female.
